# Plasma angiopoietin-2 is associated with age-related deficits in cognitive sub-scales in Ugandan children following severe malaria

**DOI:** 10.1186/s12936-020-03545-6

**Published:** 2021-01-06

**Authors:** Benson J. Ouma, Paul Bangirana, John M. Ssenkusu, Dibyadyuti Datta, Robert O. Opoka, Richard Idro, Kevin C. Kain, Chandy C. John, Andrea L. Conroy

**Affiliations:** 1 Department of Medical Microbiology, College of Health Sciences, Makerere University, Kampala, Uganda; 2 Department of Psychiatry, College of Health Sciences, Makerere University, Kampala, Uganda; 3 Department of Epidemiology and Biostatistics, College of Health Sciences, Makerere University, Kampala, Uganda; 4 Ryan White Center for Pediatric Infectious Disease and Global Health, Indiana University School of Medicine, Indianapolis, IN USA; 5 Department of Paediatrics and Child Health, Makerere University College of Health Sciences, Kampala, Uganda; 6 Centre of Tropical Medicine and Global Health, University of Oxford, Oxford, UK; 7 Department of Medicine, University of Toronto and University Health Network, Toronto, Canada

**Keywords:** Angiopoietin-2, Severe malaria, Cognition, Children

## Abstract

**Background:**

Elevated angiopoietin-2 (Angpt-2) concentrations are associated with worse overall neurocognitive function in severe malaria survivors, but the specific domains affected have not been elucidated.

**Methods:**

Ugandan children with severe malaria underwent neurocognitive evaluation a week after hospital discharge and at 6, 12 and 24 months follow-up. The relationship between Angpt-2 concentrations and age-adjusted, cognitive sub-scale z-scores over time were evaluated using linear mixed effects models, adjusting for disease severity (coma, acute kidney injury, number of seizures in hospital) and sociodemographic factors (age, gender, height-for-age z-score, socio-economic status, enrichment in the home environment, parental education, and any preschool education of the child). The Mullen Scales of Early Learning was used in children < 5 years and the Kaufman Assessment Battery for Children 2nd edition was used in children ≥ 5 years of age. Angpt-2 levels were measured on admission plasma samples by enzyme-linked immunosorbent assay. Adjustment for multiple comparisons was conducted using the Benjamini–Hochberg Procedure of False Discovery Rate.

**Results:**

Increased admission Angpt-2 concentration was associated with worse outcomes in all domains (fine and gross motor, visual reception, receptive and expressive language) in children < 5 years of age at the time of severe malaria episode, and worse simultaneous processing and learning in children < 5 years of age at the time of severe malaria who were tested when ≥ 5 years of age. No association was seen between Angpt-2 levels and cognitive outcomes in children ≥ 5 years at the time of severe malaria episode, but numbers of children and testing time points were lower for children ≥ 5 years at the time of severe malaria episode.

**Conclusion:**

Elevated Angpt-2 concentration in children with severe malaria is associated with worse outcomes in multiple neurocognitive domains. The relationship between Angpt-2 and worse cognition is evident in children < 5 years of age at the time of severe malaria presentation and in selected domains in older years.

## Background

*Plasmodium falciparum* accounts for significant global mortality, with an estimated 228 million cases and 405,000 deaths reported in 2018 [1]. Severe malaria is a leading cause of acquired neurodisability in African children [2]. Clinical complications associated with worse cognitive outcomes in severe malaria include coma [3], multiple convulsions [3], severe anaemia [4] and acute kidney injury (AKI) [5].

The angiopoietin-Tie-2 pathway is a critical regulator of endothelial and blood–brain-barrier integrity [6]. Angiopoietin (Angpt)-2 is rapidly released from activated endothelium where it inhibits Angpt-1 mediated phosphorylation of Tie-2, sensitizing endothelium to TNF, increasing expression of cellular adhesion molecules, and promoting vascular permeability [7, 8]. There is emerging evidence of Angpt-2 destabilizing endothelium through Tie-2 independent integrin-signalling leading to astrocyte and pericyte apoptosis [9, 10]. Elevated Angpt-2 is well described in severe *P. falciparum* and associated with disease severity [11–21] and mortality [11, 15–19, 21]. A relationship between elevated Angpt-2 levels and reduced cognitive function in survivors was previously reported using a summary variable that took into account multiple specific domains of cognitive and motor function [21]. It is important to know the specific cognitive and motor domains associated with elevated Angpt-2 levels, as this gives a more precise idea of brain areas affected, and areas in which to look for improvement after interventions to decrease Angpt-2 levels. For this reason, additional analyses were conducted to evaluate the relationship between Angpt-2 levels and individual cognitive and motor domains in children who survived severe malaria. Since cognitive assessment tools differ in children < 5 years of age compared to children ≥ 5 years of age, analyses are presented stratified by age.

## Methods

### Study design

Children aged 18 months to 12 years were prospectively enrolled between 2008 and 2013 at Mulago National Referral and Teaching Hospital in Kampala, Uganda in a study evaluating cognitive function after two major forms of severe malaria: cerebral malaria (CM) or severe malarial anaemia (SMA) [4]. All children with severe malaria had *P. falciparum* identified by blood smear and met case definitions for CM (Blantyre Coma Score ≤ 2; *P. falciparum* on blood smear; no known cause of coma) or SMA (*P. falciparum* on blood smear; haemoglobin level ≤ 5 g/dL) [4]. Exclusion criteria for all children included known chronic illness (including sickle cell anaemia) requiring medical care; known developmental delay; or, prior history of coma, head trauma, hospitalization for malnutrition, or cerebral palsy. Community children (CC) were enrolled to generate population-specific, age-adjusted, cognitive z-scores. Additional exclusion criteria for CC included the following: illness requiring medical care within the previous 4 weeks; or, major medical or neurological abnormalities on screening physical examination. Children were enrolled if they met criteria for CM or SMA, but were also assessed for other severe malaria criteria, including deep breathing, repeated convulsions (≥ 2 convulsions), prostration, shock, bleeding, jaundice, hypoglycaemia (glucose < 2.2 mmol/L), lactic acidosis (lactate > 5 mmol/L), AKI (defined using the Kidney Disease: Improving Global Outcomes guidelines) [22], and hyperparasitaemia (parasitaemia > 250,000 parasites/uL) [23]. Admission plasma samples were stored at − 80 °C. Angpt-2 was measured by ELISA in EDTA anti-coagulated plasma samples (DuoSet, R&D Systems, Minneapolis, MN, USA) [21].

All children underwent a medical history, physical examination and laboratory testing on enrolment. Emotional stimulation in the home was measured using age-appropriate versions of the Home Observation for the Measurement of the Environment [24]. Socio-economic status was measured as previously described [25].

### Cognitive assessments

Children with severe malaria had cognition assessed one week after discharge and at 6, 12 and 24 months follow-up by trained neuropsychological testers. Age-adjusted z-scores for cognitive outcomes were computed using the scores of the reference population of CC as previously described (4). Z-scores have average of 0 and SD 1 in the reference population over all time points. Over the course of the study 132 children crossed age groups and switched from being tested with tools designed for children < 5 years of age to tools designed for children ≥ 5 years of age.

In children < 5 years of age cognition was assessed using the Mullen Scales of Early Learning (MSEL), which measures gross motor functions, visual reception, fine motor skills, receptive language, and expressive language [26]. The four cognitive scales (visual reception, fine motor, receptive language, expressive language) are combined to generate the early learning composite which is a measure of cognitive ability. All five cognitive scales were assessed in this study. The fine motor scale assesses visual motor ability. The visual reception scale measures performance in processing visual patterns, in particular visual processing and visual memory. The receptive language scale measures a child’s ability to process linguistic input, mainly in auditory comprehension and auditory memory. The expressive language scale measures the child’s ability to use language productively through speaking ability and language formation. In children ≥ 5 years, cognition was assessed using the Kaufman Assessment Battery for Children, 2nd edition (KABC-2) [27] and the sub-scales evaluated were planning (executive reasoning), learning (immediate and delayed memory), sequential processing (short-term memory) and simultaneous processing (visual-spatial processing and problem solving). The MSEL [4] and KABC-2 [28] have been used in Ugandan children.

### Statistical analysis

Data were analysed using Stata v14.0 (StataCorp. 2015) and GraphPad Prism v7.03. Data were presented as mean (standard deviation, SD) or median (interquartile range, IQR) as indicated. Differences in median levels of Angpt-2 across age groups were evaluated using the Wilcoxon rank-sum test. Linear mixed effects modelling was used to assess the association between Angpt-2 and longitudinal changes in cognitive z-scores where observations within subject were correlated using a subject-specific intercept, and time points treated as categorical variables [21]. Models adjusted for age, gender, height-for-age z-scores, socio-economic status, enrichment in the home environment, parental education, preschool education, and disease severity on admission (presence of coma, number of convulsions, AKI) [21]. Adjustment for multiple comparisons was conducted using the Benjamini–Hochberg Procedure of False Discovery Rate.

## Results

### Demographic characteristics of the study population of children with severe malaria

**Fig. 1:**
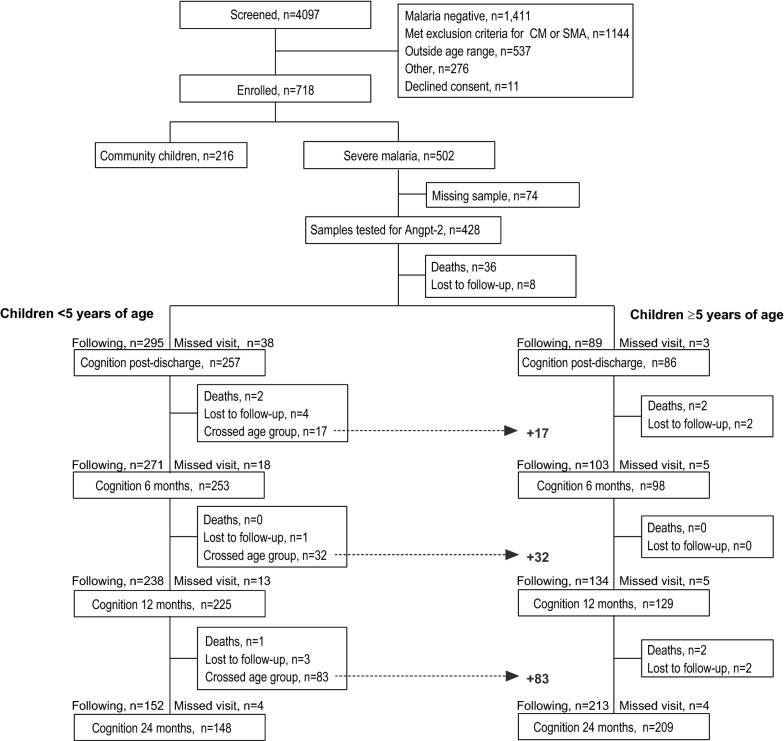
Flow chart of the study population. Children admitted to hospital with severe malaria were followed for 24 months with cognitive evaluations one-week post-discharge and at 6, 12 and 24 months follow-up. At each visit, children < 5 years of age were tested using the Mullen Scales of Early Learning, and children ≥ 5 years of age were tested using the Kaufman Assessment Battery for Children, 2nd Edition. Overall, 132 children were < 5 years of age at the time they were admitted to hospital with severe malaria and enrolled in the study and were initially tested using the Mullen Scales of Early Learning and subsequently crossed into the age group ≥ 5 years of age. Of the 132 children who crossed age groups, 129 were tested with the Kaufman Assessment Battery for Children, 2nd Edition. The number of children crossing age groups at each follow-up visit is depicted by the dotted line at each time point

A description of the 384 children included in the analysis is shown in Fig. 1. A description of the characteristics of the population, including the CC, have been described previously [4]. Overall, the median age of children enrolled in the study was 3.31 (IQR, 2.29 to 4.75) years of age and 60.4% of children were male. The prevalence of HIV was 2.4% (n = 9/382) and 4.2% (n = 16/384) of children with severe malaria had sickle cell anaemia diagnosed by genotyping after study completion [29]. The demographic characteristics and frequency of severe malaria complications in the population stratified by age at enrolment are shown in Table 1. There were no differences in median Angpt-2 levels at admission in children based on age group (< 5 years or ≥ 5 years) (p = 0.451, Table 1).

**Table 1:** Characteristics of children with severe malaria based on age at first cognitive assessment

	Children < 5 years (n = 295)	Children ≥ 5 years of age (n = 89)
Admission characteristics		
Age (years), mean (SD)	2.9 (0.9)	6.5 (1.6)
Gender (male), n (%)	172 (58.3)	60 (67.4)
Weight-for-age z-score, mean (SD)	− 1.2 (1.1)	− 1.1 (1.0)
Height-for-age z-score, mean (SD)	− 1.3 (1.5)	− 0.8 (1.2)
Socio-economic status, mean (SD)	9.1 (3.0)	10.1 (3.2)
HOME z-score, mean (SD)	− 0.08 (1.04)	− 0.08 (0.88)
Maternal education, n (%)		
Primary 6 or lower	114 (38.6)	29 (32.6)
Primary 7	63 (21.4)	20 (22.5)
Secondary or higher	109 (37.0)	36 (40.5)
Unknown	9 (3.1)	4 (4.5)
Paternal education, n (%)		
Primary 6 or lower	62 (21.0)	10 (11.2)
Primary 7	44 (14.9)	18 (20.2)
Secondary or higher	133 (45.1)	41 (46.1)
Unknown	56 (19.0)	20 (22.5)
HIV infected, n (%)	6 (2.1)	3 (3.4)
Sickle cell status, n (%)		
HbAA	286 (97.3)	78 (87.6)
HbAS	2 (0.7)	1 (1.1)
HbSS	6 (2.0)	10 (11.2)
Admission severe malaria criteria		
Cerebral malaria, n (%)	145 (49.2)	52 (58.4)
Deep breathing, n (%)	19 (6.4)	2 (2.3)
Repeated convulsions, n (%)	102 (34.6)	27 (30.3)
Prostration, n (%)	215 (72.9)	63 (70.8)
Shock, n (%)	–	–
Bleeding, n (%)	2 (0.7)	1 (1.1)
Jaundice, n (%)	197 (66.8)	51 (57.3)
Severe malarial anaemia, n (%)	187 (63.4)	40 (44.9)
Hypoglycaemia on admission, n (%)	19 (6.6)	1 (1.2)
Lactic acidosis, n (%)	117 (42.2)	19 (24.1)
Acute kidney injury, n (%)	102 (34.6)	28 (31.5)
Hyperparasitaemia, n (%)	54 (19.0)	12 (13.6)
Number of SM criteria present, mean (SD)	3.9 (1.6)	3.3 (1.2)

Neurocognitive follow-up visits, n tested	Children < 5 years	Children ≥ 5 years
One week post-discharge, n tested	257	86
Six months follow-up, n tested	253	98
12 months follow-up, n tested	225	129
24 months follow-up, n tested	148	209
Total for any follow-up visits, n tested	295	218
Admission Angpt-2 (median, IQR), ng/mL	1.78 (0.99, 3.13)	1.65 (0.84, 3.06)

Data presented as mean (standard deviation), or n (%) unless otherwise indicated

During the study period, 132 children < 5 years of age at the time of the severe malaria episode crossed into the older age group and 129 were subsequently tested using the KABC-2 (Fig. 1, Table 1). The age stratification in this study reflects the age range of the cognitive assessment tools and the age-adjusted z-scores for each cognitive and motor domain are presented in Table 2. There were 89 children ≥ 5 years of age at enrolment who were exclusively tested with the KABC-2 over follow-up. The number of children crossing age groups at each time point is depicted in Fig. 1.

**Table 2:** Mean age-adjusted z-scores across time points in children with Angpt-2 levels measured at admission

Follow-up assessment	Age adjusted z-scores for children < 5 years of age					Age adjusted z-scores for children ≥ 5 years of age			
	Fine motor	Gross motor	Receptive language	Expressive language	Visual reception	Planning	Learning	Sequential processing	Simultaneous processing
1 week	− 0.48 (1.09)	− 0.78 (1.36)	− 0.67 (1.22)	− 0.67 (1.06)	− 0.70 (1.20)	0.12 (0.89)	− 0.46 (0.97)	− 0.28 (1.28)	− 0.06 (0.96)
6 months	− 0.56 (0.99)	− 0.53 (1.25)	− 0.63 (1.13)	− 0.63 (1.06)	− 0.66 (1.14)	0.004 (0.96)	− 0.13 (1.07)	− 0.26 (1.32)	− 0.02 (1.12)
12 months	− 0.47 (1.08)	− 0.36 (1.21)	− 0.58 (1.05)	− 0.64 (1.10)	− 0.64 (1.19)	0.14 (1.02)	− 0.10 (1.06)	− 0.26 (1.24)	0.07 (1.18)
24 months	− 0.40 (0.91)	− 0.12 (0.94)	− 0.72 (0.92)	− 0.62 (0.98)	− 0.49 (1.20)	0.14 (0.75)	− 0.29 (1.09)	− 0.38 (1.25)	− 0.21 (1.02)

Data presented are mean (SD) of age-adjusted z-scores

### Angiopoietin-2 and cognitive sub-scales in survivors of severe malaria < 5 years of age at the time of the severe malaria episode

To evaluate the relationship between Angpt-2 and cognition, Angpt-2 levels at admission were compared to MSEL scores at any time point and to KABC-2 scores at any time point in which testing was done in the same child. In children < 5 years of age at the time of severe malaria episode, increased Angpt-2 levels were associated with worse cognitive scores in all domains tested (Table 3). The strongest association between increased Angpt-2 and worse z-scores for any cognitive domain was seen for visual reception (β, 95% CI − 0.41, − 0.67 to − 0.15) followed by expressive language (β, 95% CI − 0.32, − 0.56 to − 0.07), fine motor skills (β, 95% CI − 0.30, − 0.53 to − 0.06) and receptive language (β, 95% CI − 0.31, − 0.56 to − 0.06).

**Table 3:** Association between Angpt-2 level and longitudinal age-adjusted cognitive z-scores over two years follow-up in children with a severe malaria episode at < 5 years of age

	N (obs), N	Unadjusted LME Model^1^		Adjusted LME Model^1,2^		
		Beta (95% CI)	p	Beta (95% CI)	p	Significant^†^
Children < 5 years of age at time of testing (MSEL)						
Fine motor	935, 295	− 0.34 (− 0.59, − 0.09)	0.0087	− 0.30 (− 0.53, − 0.06)	0.0131	†
Gross motor	944, 295	− 0.33 (− 0.60, − 0.07)	0.0140	− 0.28 (− 0.55, − 0.02)	0.0338	†
Receptive language	935, 295	− 0.42 (− 0.68, − 0.16)	0.0016	− 0.31 (− 0.56, − 0.06)	0.0149	†
Expressive language	915, 294	− 0.29 (− 0.55, − 0.04)	0.0234	− 0.32 (− 0.56, − 0.07)	0.0120	†
Visual reception	932, 295	− 0.43 (− 0.70, − 0.16)	0.0021	− 0.41 (− 0.67, − 0.15)	0.0021	†
Children ≥ 5 years of age at time of testing (KABC-2)						
Planning^3^	**–**			**–**	**–**	
Learning	186, 129	− 0.43 (− 0.76, − 0.10)	0.0104	− 0.39 (− 0.74, − 0.05)	0.0253	†
Sequential processing	186, 129	− 0.38 (− 0.84, 0.07)	0.0992	− 0.27 (− 0.72, 0.17)	0.2302	
Simultaneous processing	182, 127	− 0.19 (− 0.48, 0.10)	0.2018	− 0.34 (− 0.61, − 0.08)	0.0101	†

^†^p < 0.05 in model after correction for multiple comparisons (n = 5 for children < 5 years of age; n = 3 for children ≥ 5 years of age)

^1^Linear mixed effects (LME) model where observations within subject were correlated using a subject-specific intercept, and time points as categorical variables

^2^Models included age, gender, height-for-age z-score, socio-economic status, home environment, parental education, preschool education of the child (any), presence of coma, number of seizures during admission, and acute kidney injury

^3^Planning is assessed in children ≥ 7 years of age, so results are not available for children < 5 years of age at the time of the severe malaria episode

### Angiopoietin-2 and cognitive sub-scales in survivors of severe malaria based on clinical syndrome at presentation

To evaluate whether the relationship between Angpt-2 and cognitive outcomes was affected by the presence of neurological complications on admission, analyses were stratified based on the study group: CM or SMA (Table 4). Angpt-2 levels were associated with worse cognitive scores in multiple domains in children < 5 years of age at the time of severe malaria with worse z-scores in fine motor skills, receptive language and visual reception in children with SMA and worse z-scores in gross motor skills, receptive and expressive language, and visual reception in children with CM (Table 4). There was a trend towards persistent differences in learning in children < 5 years of age at the time of severe malaria episode who were subsequently tested using the KABC-2 in children with CM (Table 4). However, there was limited power to detect differences in children ≥ 5 years of age tested using the KABC-2, especially after stratifying by study group on admission.

**Table 4:** Association between Angpt-2 level and longitudinal age-adjusted cognitive z-scores over two years follow-up in children based on the severe malaria group at presentation in children < 5 years of age

	Adjusted LME estimates in SMA^1,2^				Adjusted LME estimates in CM^1,2^			
	N (obs), N	Beta (95% CI)	P	Significant †	N (obs), N	Beta (95% CI)	P	Significant †
Children < 5 years of age at time of testing (MSEL)								
Fine motor	476, 148	− 0.34 (− 0.58, − 0.09)	0.0078	†	459, 147	− 0.35 (− 0.79, 0.08)	0.110	
Gross motor	484, 148	− 0.25 (− 0.57, 0.08)	0.132		460, 147	− 0.51 (− 0.95, − 0.07)	0.0229	†
Receptive language	480, 148	− 0.40 (− 0.69, − 0.10)	0.0096	†	455, 147	− 0.55 (− 0.98, − 0.11)	0.0141	†
Expressive language	470, 148	− 0.23 (− 0.51, 0.06)	0.116		445, 146	− 0.65 (− 1.07, − 0.23)	0.0029	†
Visual reception	479, 148	− 0.38 (− 0.70, − 0.05)	0.0235	†	453, 147	− 0.65 (− 1.09, − 0.21)	0.0043	†
Children ≥ 5 years of age at time of testing (KABC-2)								
Planning^3^	**–**					**–**	**–**	
Learning	139, 36	0.02 (− 0.62, 0.67)	0.9404		204, 53	− 0.63 (− 1.35, 0.10)	0.0880	
Sequential processing	139, 36	0.09 (− 0.83, 1.02)	0.8339		205, 53	0.05 (− 0.84, 0.93)	0.9176	
Simultaneous processing	138, 36	− 0.43 (− 1.13, 0.27)	0.2192		205, 53	− 0.25 (− 0.96, 0.46)	0.4759	

^†^p < 0.05 in model after correction for multiple comparisons (n = 5 for children < 5 years of age; n = 3 for children ≥ 5 years of age)

^1^Linear mixed effects (LME) model where observations within subject were correlated using a subject-specific intercept, and time-points as categorical variables

^2^Models included age, gender, height-for-age z-score, socio-economic status, home environment, parental education, preschool education of the child (any), number of seizures during admission, and acute kidney injury

^3^Planning is assessed in children ≥ 7 years of age, so results are not available for children < 5 years of age at the time of the severe malaria episode

### Angiopoietin-2 and cognitive sub-scales in survivors of severe malaria in children ≥ 5 years of age at time of severe malaria episode

Another critical question is whether there is an age-specific window of susceptibility between Angpt-2 and cognition based on the child’s age at the time of the severe malaria admission. The data from the MSEL, presented above, showed associations of elevated Angpt-2 with worse scores in multiple domains in children < 5 years of age at time of severe malaria episode, and the effects persisted with differences in learning and simultaneous processing as children crossed age groups and were tested using the KABC-2 (Table 3).

The KABC-2 analysis was repeated in 89 children who were ≥ 5 years of age at study enrolment (Table 5). In children ≥ 5 years at the time of the severe malaria episode, there were no significant relationships between Angpt-2 and cognitive sub-scales (p > 0.05 for all, Table 5).

**Table 5:** Association between Angpt-2 level and longitudinal age-adjusted z scores over two years follow-up in children with a severe malaria episode at ≥ 5 years of age

	N (obs), N	Unadjusted LME Model^1^		Adjusted LME Model^1,2^		
		Beta (95% CI)	P	Beta (95% CI)	P	Significant †
Children ≥ 5 years of age at time of severe malaria episode (enrolment time point) (KABC-2)						
Planning^3^	194, 84	− 0.13 (− 0.42, 0.16)	0.3783	− 0.16 (− 0.52, 0.20)	0.3844	
Learning	343, 89	− 0.22 (− 0.63, 0.18)	0.2764	− 0.26 (− 0.72, 0.20)	0.2682	
Sequential processing	344, 89	− 0.10 (− 0.60, 0.40)	0.6967	− 0.01 (− 0.59, 0.56)	0.9644	
Simultaneous processing	343, 89	− 0.21 (− 0.59, 0.16)	0.2659	− 0.20 (− 0.62, 0.22)	0.3457	

^†^p < 0.05 in model after correction for multiple comparisons (n = 4 for children ≥ 5 years of age at enrolment)

^1^Linear mixed effects (LME) model where observations within subject were correlated using a subject-specific intercept, and time points as categorical variables

^2^ Models included age, gender, height-for-age z-score, socioeconomic status, home environment, parental education, preschool education of the child (any), presence of coma, number of seizures during admission, and acute kidney injury

^3^Planning is assessed in children ≥ 7 years of age

## Discussion

In this study, elevated Angpt-2 levels in Ugandan children with severe malaria were associated with lower z-scores over two-years follow-up in visual reception, fine motor development, and receptive and expressive language in children < 5 years of age at the time of testing, and in lower learning and simultaneous processing z-scores in children ≥ 5 years of age at the time of testing, but the latter associations are seen only in children < 5 years of age at the time of the severe malaria episode. These results suggest that Angpt-2 is associated with worse cognitive scores in multiple domains in children with severe malaria who are < 5 years of age at time of severe malaria, and these problems persist into older age, but that Angpt-2 is not associated with worse cognitive outcomes in any domain in children who are ≥ 5 years of age at the time of severe malaria infection.

Assessment of the effects of age on the relationship to cognitive outcomes is made more difficult by the change in age status of many children over follow-up, such that a number of children initially tested at < 5 years with the MSEL became 5 years of age during the study and switched to testing with the KABC-2. Thus, the results for the MSEL testing are from children who were all under 5 years at the time of severe malaria episode, while the results for KABC-2 testing include both children who were < 5 years at the time of severe malaria exposure as well as children ≥ 5 years at the time of severe malaria exposure. Further analysis of relationships between Angpt-2 and KABC-2 based on child age at malaria exposure showed the relationships between Angpt-2 and poorer learning and simultaneous processing was driven by children < 5 years of age at enrolment. There were no significant associations between Angpt-2 and KABC-2 results in children already ≥ 5 years at enrolment. Although the number of children ≥ 5 years at enrolment (n = 89) was lower than the number of children < 5 years of age at enrolment who subsequently became 5 years of age (n = 129) and were tested by the KABC-2, this does not seem to be the primary explanation for the lack of association in older children, as effect sizes were lower in children ≥ 5 years at the time of the severe malaria episode. However, it is possible that with a larger sample size, some associations seen might have reached statistical significance.

The relationship between Angpt-2 across cognitive domains in children < 5 years of age but not in children ≥ 5 years of age is consistent with the ‘early vulnerability’ perspective showing worse cognitive outcomes after brain injury at an earlier age versus later childhood [30, 31]. These studies suggest that central nervous system (CNS) injury during severe malaria occurring earlier in childhood (< 5 years), when cognitive abilities are not yet developed or are developing, results in widespread disruption in function compared to injury in later childhood. In children who were < 5 years at the time of severe malaria, but ≥ 5 years of age at testing, Angpt-2 levels were related to worse outcomes in learning and in simultaneous processing, which is related to visual-spatial ability. Thus, children < 5 years of age with severe malaria show an association of elevated Angpt-2 levels and impaired visual-spatial abilities that persists as they get older. Of note, it is not possible to rule out an association between Angpt-2 and worse cognitive outcomes in older children as the number of children who were ≥ 5 years of age at severe malaria exposure was limited. The age stratification at 5 years in this study reflects the availability of cognitive assessment tools as there was not a single battery that could assess cognition over the entire age range of the study (i.e., 18 months to 12 years). However, the cut-off of 5 years of age is arbitrary and may not accurately reflect periods of age-related vulnerability. Additional studies are needed to understand the long-term impact of age-related susceptibilities in cognition and how early injury relates to long-term deficits.

A relationship between Angpt-2 and blood–brain-barrier dysfunction was previously reported in this cohort of children with CM [21]. Angpt-2 could contribute to cognitive impairment through increases in blood–brain-barrier permeability, leading to increased neuro-active metabolites in the cerebrospinal fluid and consequent brain parenchymal injury [21]. There is also evidence that Angpt-2-integrin interactions can lead to astrocyte and pericyte apoptosis in experimental models of hyperglycaemia [9, 10]. Both astrocytes and pericytes are involved in active regulation of blood–brain-barrier integrity and loss of either cell type in conjunction with endothelial activation could contribute to blood–brain-barrier dysfunction and neuro-inflammation in malaria (reviewed in [32]). Additional studies are needed to determine whether Angpt-2-integrin interactions contribute to endothelial activation and blood–brain-barrier dysfunction in severe malaria, and whether maturational changes in the blood–brain-barrier explain the Angpt-2 age-related effects observed in this study.

In this cohort, Angpt-2 levels were comparable in children with CM and SMA [21], and in the present study, there were significant associations between Angpt-2 and cognition in children with CM and SMA. These results suggest a common mechanism of brain injury in children with CM and SMA where parasite sequestration in the microvasculature leads to vascular congestion, tissue hypoxia and local inflammatory responses resulting in increased Angpt-2 expression and blood–brain-barrier dysfunction [33]. An increase in vascular permeability may facilitate parasite antigens like histidine-rich protein-2 crossing the blood–brain-barrier [34], resulting in an increase in neuro-inflammation [35, 36], neuro-active metabolites [37], and axonal injury [38]. Other bioactive parasite products (i.e., parasite histones) may also contribute to vascular thrombosis and vascular leak [39]. Increased availability of more advanced magnetic resonance imaging (MRI) techniques in low-and-middle-income settings may yield important insights into structural and functional brain changes across the spectrum of uncomplicated malaria, to severe (non-cerebral malaria) and cerebral malaria [40]. Although most radiological studies have been conducted in the context of cerebral malaria [41], a study including adults with non-cerebral severe malaria observed diffuse brain swelling by MRI that was not specific to coma [40], suggesting sub-clinical brain swelling may occur in severe malaria even in the absence of neurological signs. A recent study comparing brain MRI findings with blood-retinal barrier integrity and neurological outcomes identified vascular leak and retinal whitening, a sign of tissue hypoxia, with the development of neurologic deficits in survivors [42]. As Angpt-2 is induced in conditions of hypoxia, sensitizes the endothelium to inflammation and is an important regulator of vascular permeability, these findings lend additional support to vascular dysfunction being a risk factor for increased mortality as well as neurocognitive injury.

## Conclusion

In this prospective, longitudinal study, elevated Angpt-2 was associated with worse neurocognitive outcomes in children across multiple domains when they were exposed to severe malaria at < 5 years of age, and these associations persisted even after the age of 5 years. No association was seen between Angpt-2 levels and neurocognitive outcomes in children > 5 years of age at the time of severe malaria admission, but a larger sample size may be required to detect small associations in this age group. A long-term follow-up study is currently underway to evaluate the important question of how findings from early childhood relate to subsequent functioning across different domains (cognition, behaviour, social adjustment, academic, and economic productivity) in later childhood and adulthood.

## Data Availability

The datasets used and/or analyzed during the current study are available from the corresponding author on reasonable request.

## Abbreviations

AKI: Acute kidney injury
CNS: Central nervous system
CM: Cerebral malaria
CI: Confidence interval
CC: Community children
HIV: Human immunodeficiency virus
IQR: Interquartile range
KABC-2: Kaufmann Assessment Battery for Children 2nd edition
LME: Linear mixed effects
MSEL: Mullen Scales of Early Learning
SD: Standard deviation
SMA: Severe malarial anaemia
WHO: World Health Organization
